# Chemical Genetics: Budding Yeast as a Platform for Drug Discovery and Mapping of Genetic Pathways

**DOI:** 10.3390/molecules17089258

**Published:** 2012-08-02

**Authors:** Jorrit M. Enserink

**Affiliations:** Department of Molecular Biology, Institute of Medical Microbiology and Centre for Molecular Biology and Neuroscience, Oslo University Hospital, Sognsvannsveien 20, NO-0027 Oslo, Norway; Email: jorrit.enserink@rr-research.no; Tel.: +47-230-740-66; Fax: +47-230-740-61

**Keywords:** chemical-genetics, budding yeast, *Saccharomyces cerevisiae*, genetic interaction

## Abstract

The budding yeast *Saccharomyces cerevisiae* is a widely used model organism, and yeast genetic methods are powerful tools for discovery of novel functions of genes. Recent advancements in chemical-genetics and chemical-genomics have opened new avenues for development of clinically relevant drug treatments. Systematic mapping of genetic networks by high-throughput chemical-genetic screens have given extensive insight in connections between genetic pathways. Here, I review some of the recent developments in chemical-genetic techniques in budding yeast.

## 1. Introduction

What is chemical genetics? Chemical genetics is a research approach that makes use of small molecules to explore biological functions and processes; “chemical” indicates the use of biologically active chemicals, whereas “genetics” refers to the fact that this research approach is based on principles often used in the field of genetics. Chemical-genetic screens can be used to determine the mode of action of a given biologically active compound by identifying its cellular target. Alternatively, a drug can be designed to specifically inhibit or activate a protein of interest; this compound can subsequently be used in a high-throughput screen to identify novel functions of the target protein. Typically, a compound is used that affects the function of a single gene product in the context of a complex cellular milieu. This compound may then be employed to screen a large collection of mutants using a specific biological effect as a read-out. Many chemical-genetic studies have made use of protein-targeting drugs. However, chemical-genetic screens are not necessarily restricted to proteins, but can also be expanded to include drugs that target RNA, DNA, or even cellular metabolites. Here I will review some of the recent publications on chemical-genetic studies in the model organism *S. cerevisiae*, also known as brewer’s yeast or baker’s yeast.

## 2. History of Budding Yeast as a Model Organism

The vast majority of all chemical-genetic screens have made use of budding yeast, because yeast has several important advantages. For example, it is easy and cheap to culture and it grows fast, with a doubling time of 90–100 min under optimal conditions. It is amenable to genetic techniques, and genes can be modified or deleted within just a few days. Yeast cells can exist in a haploid and a diploid life cycle, which makes it easy to combine mutations by intercrossing. The *S. cerevisiae* genome consists of 16 chromosomes, totaling approximately 12.1 million basepairs. The first eukaryote to be completely sequenced, a map of the yeast genome was released in 1996 [1].

How did budding yeast rise to prominence? The history of budding yeast as a model organism in research basically starts with the founding of the Carlsberg brewery in 1844 in Valby, an outskirt of Copenhagen. At the time, beer brewing was mainly conducted in small, local breweries, and the quality of beer between the different batches was often inconsistent. The Carlsberg Laboratory was founded in 1875 with the aim of developing scientific and industrial methods that would lead to a more consistent quality of beer. Of particular importance to the development of yeast genetics was a microbiologist named Emil Christian Hansen working at the Carlsberg Laboratory. Previous studies by Louis Pasteur on wine fermentation had indicated that different microorganisms could be isolated during the fermentation process, some of which were thought to be responsible for producing ill-tasting byproducts that spoiled the quality of the wine. Inspired by this information, Dr. Hansen discovered how to isolate and culture individual isolates of yeast clones in 1883. The isolation of a pure yeast strain was an important step for beer brewing, but it was also a major advancement for science. 

A second key development in yeast research came almost 50 years later, when Dr. Øjvind Winge (also working at the Carlsberg Laboratory) discovered that yeast cells can alternate between haploid and diploid life cycles; haploid cells can mate to form a diploid cell, which subsequently can undergo meiosis to form four haploid cells, each of which can be isolated for phenotypic analysis. He then showed that many genetic traits, such as colony morphology and fermentation markers, follow simple Mendelian rules. These discoveries laid the foundation for the field of classical yeast genetics.

## 3. Classical Genetics

Classical yeast genetics refers to the genetic techniques commonly used before the dawn of molecular biology in the mid-20th century, when the molecular nature of genes had not yet been identified. Typically, genetic screens were aimed at identifying the genes responsible for a particular phenotype, a method referred to as *forward genetics*. Forward genetic screens start with a given phenotype of interest, and large collections of mutants are then screened for mutations that specifically alter that phenotype. A well-known example is the study of eye color in *Drosophila melanogaster* by Thomas Hunt in the early 1900s [2,3]. Wild-type flies have red eyes, and Morgan and his students screened for changes in eye color in mutagenized flies, such as white and pink eyes. Crosses between mutants revealed several mutations responsible for the altered phenotype, and Morgan proposed that chromosomes form the physical basis of heredity [4], even though the concept and nature of genes remained enigmatic for several decades.

*Reverse genetics*, as the name implies, is a research approach that follows the opposite direction of forward genetics; a gene is mutated and the effect of that mutation is studied by searching for a phenotype. Thus, forward genetics is phenotype-centered and is aimed at identifying the genes responsible for that specific phenotype, whereas reverse genetics is gene-centered and is aimed at identifying the phenotype associated with a given gene. The advent of molecular biology, which resulted both in a clear definition of the concept of the gene as well as development of techniques to modify gene sequences and to modulate gene expression levels *in vivo*, has greatly stimulated reverse genetics.

Before the era of molecular biology, the classical problem was to identify genes associated with a given phenotype. Identification of these genes was often a laborious and pain-staking affair, which involved creating a library of mutants by mutagenesis, screening for mutants with an altered phenotype, and mapping of the mutations through genetic complementation studies. However, in the post-genomic era, this problem has been reversed; high-throughput genome sequencing projects have identified all the genes of numerous organisms, but the function of many of those genes remains unclear. For instance, the genome of budding yeast, arguably the best understood eukaryotic organism, contains approximately 6,000 genes, but the function of nearly 1,000 of them remains elusive [5].

## 4. Genetics in the Post-Genomic Era

**Figure 1:**
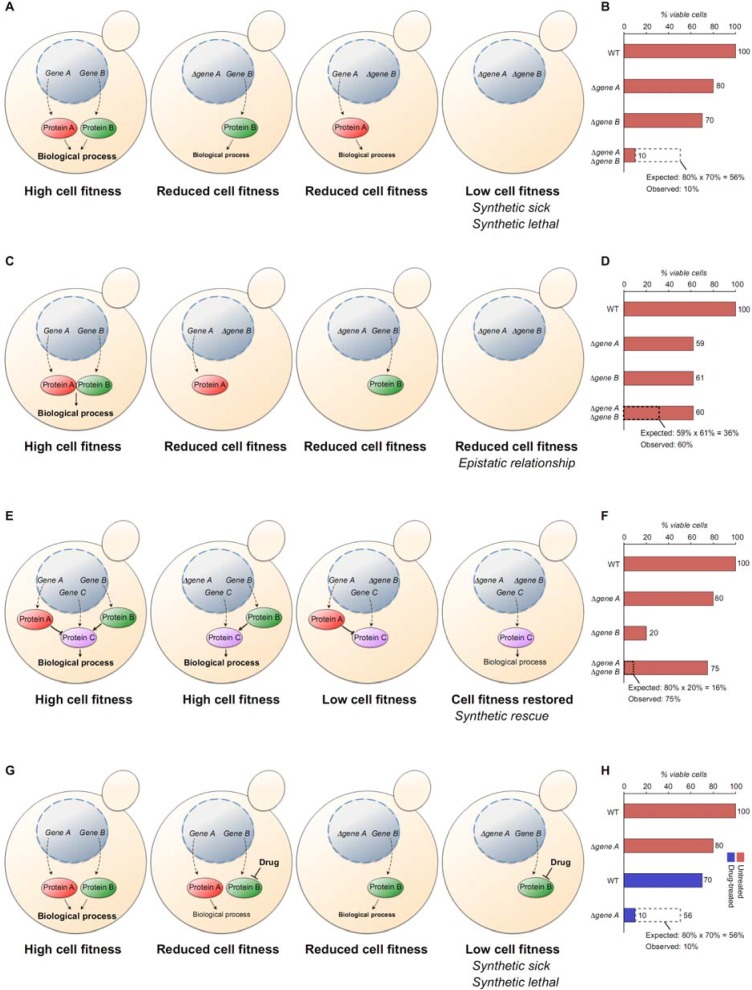
Defining different forms of genetic relationships between genes. (**A**) Negative genetic interaction; (**B**) Depiction of cell viability of the example shown in (*A*); (**C**) Epistatic genetic interaction;(**D**) Depiction of cell viability of the example shown in (*C*); (**E**) Positive genetic interaction; (**F**) Depiction of cell viability of the example shown in (*E*); (**G**) Negative chemical-genetic interaction; (**H**) Depiction of cell viability of the example shown in (*G*). See text for details.

A major tool in a geneticist’s toolbox to identify the function of a gene is the analysis of its genetic network. Genes (or more exactly, their products) do not work alone in the cellular processes they control, but rather function in intricate biological networks to fine-tune these processes in response to cellular and environmental stimuli. These biological networks tend to be robust, and removing one component often has minor consequences for cell viability because other components of the network can compensate for the lost function, an effect sometimes referred to as ‘genetic buffering’ [6,7]. However, simultaneous removal of multiple network components may diminish the process controlled by the network, with serious consequences for cell viability. This is the basis for genetic interaction (Figure 1 and see below). Thus, combining different mutations can give insight in the function of genes.

### 4.1. Defining Genetic Interactions

Various forms of genetic interaction can be defined. For instance, a negative genetic interaction occurs when the combination of two mutations results in a worse phenotype than expected based on either single mutation alone. This may happen when two redundant genes function in parallel pathways to control a certain process, and combining mutations in these genes may cripple the process and lead to reduced fitness of the yeast cell, leading to a synthetic growth defect. In the example shown in Figure 1A, two genes (gene A and gene B) encode two proteins that function in parallel pathways to regulate a specific biological process. Cells that have a wild-type copy of both genes have high fitness and are viable. 

Deleting gene A results in reduced fitness and lower cell viability because the process is carried out less efficiently, however the mutant still survives because gene B can partially compensate for loss of gene A. The reciprocal situation has the same result, *i.e.*, gene A can partially compensate for loss of gene B. However, the cell can no longer carry out the biological process when both genes are lost simultaneously, leading to a much more severe phenotype than expected based on either single mutation alone (Figure 1B). An extreme form of negative genetic interaction is synthetic lethality, in which the combination of two mutations results in cell death. An example of synthetic lethality is the combination of mutations in *RAD52* and *RAD27* [8]. Rad52 is involved in repair of DNA double strand breaks by homologous recombination, whereas Rad27 is a flap endonuclease required for Okazaki fragment processing. *rad27Δ* mutants suffer from extensive DNA replication errors and DNA double strand breaks, which are mainly processed by Rad52-dependent DNA repair; in absence of Rad52, the DNA damage that occurs in *rad27Δ* mutants is no longer repaired, leading to cell death [8]. 

When two gene products function in the same pathway to control a certain process, for example when they form a mandatory complex, the combination of mutations in these two genes will not have a phenotype that is significantly different than either single mutation alone. This is referred to as an epistatic relationship, which occurs when the phenotype of one mutation is obscured by mutation of another gene. In the example shown in Figure 1C, Gene A and gene B encode two proteins that form a complex, and these proteins can only regulate a specific process when bound to each other. Deleting either gene A or gene B results in loss of the active complex and the biological process no longer occurs. Deleting both genes simultaneously will not result in a worse phenotype (Figure 1D). For instance, the DNA double strand break repair proteins Mre11, Rad50 and Xrs2 (Nbs1 in humans) function in a mandatory complex, and the DNA damage sensitivity of *mre11Δ* *rad50Δ* double mutant cells is not worse than that of either single mutant alone [9]. 

Two mutations can also have a positive genetic interaction, when the phenotype resulting from one mutation can be rescued by additional mutation of a second gene. In the example shown in Figure 1E, Three genes A, B and C encode proteins that regulate a specific biological process. Protein C is negatively regulated by protein A and positively by protein B. The biological process will still be carried out efficiently if gene A is deleted. However, deletion of gene B will tip the balance towards inactivation of protein C by protein A, leading to inhibition of the biological process and reduced cell fitness, which may manifest itself in slow growth or cell death (Figure 1F). Several terms have been proposed to describe this phenomenon, such as synthetic rescue, synthetic suppression or phenotypic repression, all with essentially the same meaning. As an example of synthetic rescue, deletion of *SML1* rescues the lethal phenotype of a *mec1Δ* mutation [10,11]. *MEC1* is an essential gene that encodes a checkpoint kinase involved in activation of the DNA replication checkpoint in response to DNA replication stress [12], whereas *SML1* encodes an inhibitor of ribonucleotide reductase [10,11]. Increased ribonucleotide synthesis is an essential aspect of the cellular response to DNA replication stress, and Mec1 activates a signaling pathway that degrades Sml1 to induce synthesis of ribonucleotides [10,11]. In absence of Mec1, cells fail to produce sufficient ribonucleotides to survive DNA replication stress because Sml1 continuously inhibits the ribonucleotide synthase, explaining why deletion of *SML1* rescues lethality of *mec1Δ* mutants.

Finally, drugs that specifically target a protein can also be used for mapping of genetic pathways. This is particularly useful for studies of essential genes that cannot be deleted. In this case, mutants can be treated with the drug to specifically modulate the activity (either positively or negatively) of the protein of interest to study relations between genes. In the example shown in Figure 1G, Gene A and gene B encode two proteins that control a specific process (similar to the example shown in Figure 1A). Gene B is an essential gene that cannot be deleted. However, a drug has been designed to specifically inhibit its function, and treating mutant cells lacking gene A results in a synthetic growth defect or synthetic lethality, a phenotype that is significantly worse than would have been expected based on either treatment of wild-type cells with the drug or by deleting gene A (Figure 1H). Although only a negative interaction is depicted here, epistatic and positive interactions are also possible.

### 4.2. High-Throughput Systematic Screens

One of the major consequences of mapping the yeast genome has been a shift in research from a focus on individual genes to a more global view of genetic networks [13]. This was aided by the completion of the yeast deletion collection, a library of deletion mutants lacking all non-essential genes [14,15], and by the development of high-throughput methods to screen this library. During the past decade, numerous high-throughput studies have systematically screened for genetic interactions. An important methodological development was the synthetic genetic array (SGA) screen, in which a mutation in the gene of interest is crossed into the entire yeast deletion collection [16,17]. The resulting double mutants are then analyzed for genetic interactions, which can be either synthetic growth defect/lethality or synthetic suppression relationships. Originally performed manually, these screens are now usually performed in an automated format; growth of double mutants is monitored by automated imaging of the colonies followed by processing by an algorithm to calculate relative growth rates [18]. Alternative methods have been developed for automated analysis of genetic interactions, such as synthetic lethality by microarray, where genetic interactions between genes are detected and quantified by hybridizing genomic DNA of double mutants to DNA micro-arrays [19]. Together, these high-throughput systematic screens have mapped millions of relationships between genes, allowing the construction of a global genetic interaction map of yeast [20]. 

However, one problem of these high-throughput genetic screens is that essential genes are underrepresented, because they are absent from the yeast deletion collection. This problem may be circumvented by using partial-loss-of-function mutations. For instance, one study made use of a promoter replacement approach, in which the endogenous promoter of 575 essential genes was replaced with the tetracylin-repressible tet promoter [21,22]. Query mutations are then crossed into this library and the double mutants are spotted on plates containing doxycycline (a tetracyclin analog) to repress transcription of essential genes, and genetic interactions are scored by monitoring growth rates of the double mutants. Analysis of the genetic interaction network of these tet-repressible essential genes revealed that it has an interaction density five times that of non-essential genes [21], indicating that current maps of genetic networks (which are almost entirely based on screens with non-essential genes) significantly underestimate the size of the actual genetic network of the yeast genome. One potential complication of this strategy is that the expression levels of some essential genes may be more consequential for cell viability than that of other essential genes, and the concentration of doxycycline that will block growth of these tet-repressible strains will be much lower than that of other strains. Therefore, it may be challenging to titrate doxycycline to a concentration window that targets all essential genes, *i.e.*, high enough for genetic interactions to occur, but low enough to permit a level of transcription that allows growth of the tet-repressible single mutants (too much doxycycline will completely inhibit transcription of the tet-repressible essential genes, thus blocking growth of the mutant regardless of genetic interactions). 

Another popular loss-of-function mutant phenotype is temperature sensitivity, in which a mutant is viable at permissive temperature but very sick or inviable at non-permissive temperature. Most temperature-sensitive mutants display a partial loss-of-function phenotype at semi-permissive temperatures, allowing them to be used in SGA screens. Large collections of temperature-sensitive mutants of essential genes have recently been developed, paving the way for further exploration of the genetic interaction networks of essential genes [23,24]. However, the heat-shock response resulting from the elevated temperatures needed for inactivation of these alleles can have a confounding effect, potentially complicating the interpretation of such screens. Furthermore, some temperature-sensitive mutations inhibit only one specific function of a protein, with less or no effect on other functions of that protein. For example, some temperature-sensitive alleles of the cyclin-dependent kinase *CDC28* (also known as *CDK1*), which regulates the cell cycle in budding yeast [25], specifically block the function of Cdc28 early in the cell cycle (e.g., *cdc28-4*, which blocks cell cycle progression at late G1 under restrictive temperature), whereas others act late in the cell cycle (for example *cdc28-1N* and *cdc28-13*, which block the cell cycle at G2/M phase). Screening for genetic interactions with these alleles may only give information on very specific functions of *CDC28*. Therefore, while informative, there are disadvantages to the use of temperature-sensitive alleles in genome-wide screens. 

Inhibiting essential genes, either by reducing their expression levels or by temperature-sensitive inactivation, can lead to a complete loss of all the functions of the protein. This is a potential disadvantage, because enzymes can have non-catalytic functions in addition to their enzymatic activity. For example, there are numerous reports in the literature of kinase-independent roles of kinases in signal transduction; just to name a few, ERK1/2, aurora, ZAP-70, FAK and Cdk1 all have kinase-independent functions in addition to their catalytic activity [26,27,28,29,30]. Therefore, more sophisticated methods are required to be able to differentiate between kinase-dependent and kinase-independent functions of kinases, such as chemical-genetics. 

### 4.3. High-Throughput Chemical-Genetic Screens

A powerful chemical-genetic tool has been developed to specifically inhibit the catalytic activity of kinases without affecting their expression levels [31]. This strategy exploits the gatekeeper residue (generally a conserved hydrophobic amino acid) in the ATP binding site of the kinase. The ATP binding pocket can be modified by substituting the gatekeeper residue with a smaller amino acid like glycine or alanine, such that it can accommodate a sterically bulky ATP analog that is inhibitory to kinase activity [31]. Because of its bulkiness, it cannot bind and inhibit any of the other kinases in the cell, and therefore this approach is highly specific. One example of an engineered, analog-sensitive kinase allele is *cdc28-as1*, in which the gatekeeper residue (a phenylalanine at position 88) has been replaced with a glycine, such that the Cdc28-as1 protein can be inhibited with the bulky ATP analog 1-NM-PP1 [31].

*CDC28* is an essential gene, and therefore it cannot simply be deleted to study its genetic network. However, titration of 1-NM-PP1 gradually inhibits growth of *cdc28-as1* mutants, and sub-lethal doses of 1-NM-PP1 that reduce the growth rate of *cdc28-as1* mutants have been used to screen for mutations that aggravate the phenotype of the *cdc28-as1* allele [32]. This screen revealed numerous novel functions of Cdc28, in particular in regulation of the basal transcription machinery and cell cycle progression [32,33,34]. This strategy can be used for any other kinase to specifically explore kinase-dependent functions. 

By far most of the high-throughput genetic screens have been performed under conditions of steady-state loss-of-function (by using deletion mutants as a query, or continuous reduction of activity of a specific protein). While informative, the cells may adapt to the cellular conditions under which these query genes are inactive. One advantage of a chemical-genetic approach is that the consequence of just briefly inhibiting the kinase of interest can now be investigated. This will be particularly interesting when combined with specific cellular challenges, like nutrient starvation and DNA damage.

Of course, chemical-genetic methods are not restricted to just mapping of genetic networks, but can also be used to characterize the biochemical functions of a given protein. Numerous studies have used chemical-genetic methods to identify novel protein functions, and here I will only highlight a few recent reports.

Gatekeeper mutations are particularly suitable for identification of kinase targets. For example, by performing *in vitro* kinase assays with Cdc28-as1 on an arrayed library of affinity tag-coupled yeast proteins, numerous potential Cdc28 targets were identified [35]. Many of these substrates were also identified in a combined chemical-genetic/proteomic approach that mapped Cdc28-dependent phosphorylation sites *in vivo* [36]. These studies predicted many putative Cdc28 targets, including the DNA damage checkpoint protein Rad9. Furthermore, in a chemical-genetic screen *CDC28* was previously found to have extensive genetic interactions with DNA damage checkpoint genes [37], indicating that Cdc28 may have a function in regulation of the checkpoint. Indeed, a recent study found that Rad9 is a *bona fide* Cdc28 target, and that phosphorylation of Rad9 promotes checkpoint activity [38]. Thus, chemical-genetic screens to map genetic networks of kinases, in combination with chemical-genetic biochemical methods to predict and verify kinase targets, are a powerful tool to identify novel signal transduction pathways and networks.

Gatekeeper mutations have also been used to study regulation of transcription. For example, several studies have used the *kin28-as1* gatekeeper allele to probe the function of Kin28 in this process. Kin28 is homologous to human CDK7, a cyclin dependent kinase that phosphorylates the C-terminal domain (CTD) of RNA polymerase II [39]. The CTD consists of 26 (52 in humans) heptameric repeats of the sequence Y_1_S_2_P_3_T_4_S_5_P_6_S_7_, and Kin28 preferentially targets Serine at position 5. Most studies have used temperature-sensitive alleles of *KIN28* and found that inactivation of Kin28 has major consequences for transcription [39,40,41]. However, recent chemical-genetic studies that used analog-sensitive *kin28-as1* mutants have shown that the kinase activity of Kin28 plays a much more subtle role in transcription than expected based on results from studies that used temperature-sensitive *kin28* alleles, showing that only a subset of genes is affected by Kin28 kinase activity [42,43,44]. Rather, these chemical-genetic studies found that Kin28 kinase activity appears to be important for mRNA capping [44,45].

Another example of the identification of an unexpected function of a protein comes from a chemical-genetic study of Cdc7. Cdc7 is a kinase best known for its role in initiation of DNA replication [46]. However, chemical-genetic analysis of the function of Cdc7 (using the analog-sensitive *cdc7-as* allele) revealed that Cdc7 kinase activity also promotes meiotic progression by enabling transcription of *NDT80*, a meiosis-specific transcription factor, and that Cdc7 is required for proper orientation of sister kinetochores [47]. 

As a final example, a recent variant of a chemical-genetic screen aimed to characterize the pathways that control cell-surface localization of the arginine permease Can1 [48]. Can1 normally transports arginine from the extracellular environment, but it can also import the toxic arginine analog canavanine. By screening the yeast deletion collection for mutations that conferred resistance to canavanine (due to defects in plasma membrane localization of Can1), numerous proteins were found to be involved in transport of Can1 to and from the cell membrane, several of which not previously known to be involved in this process [48].

Taken together, these examples illustrate the power of chemical-genetics in characterization of very diverse cellular processes.

## 5. Drug Target Identification and Chemical Profiling of the Yeast Genome

Many compounds are known to have biological effects even though the molecular targets of these compounds often remain unknown. Identification of the cellular targets of bioactive compounds is a major bottleneck in the development of novel therapeutic agents [49]. Budding yeast has been instrumental in unraveling the mode of action of numerous compounds. One well-known example is the discovery of the cellular targets of the macrolide rapamycin during the early 1990s. Rapamycin is a secondary metabolite produced by the bacterium *Streptomyces hygroscopicus,* and is currently used as an immunosuppressive after organ transplantation [50]. Rapamycin also has potent antifungal activity [51,52], and a screen for budding yeast mutants that had become resistant to rapamycin revealed three genes required for rapamycin toxicity, *i.e.*, *FPR1*, *TOR1* and *TOR2* [53,54]. Fpr1 is a proline isomerase essential for rapamycin toxicity, whereas Tor1 and Tor2 are two serine/threonine kinases with high sequence similarity; upon binding to rapamycin, Fpr1 sequesters Tor1/2 in an inactive complex. mTOR was later identified to be the mammalian homolog of Tor1/2 [55]. Thus, budding yeast can be used to identify the mode of action of compounds that have clinical relevance.

The research field of chemical-genomics aims to identify functional relationships between specific genes and chemical compounds through systematic analysis of all genes in a genome [49]. The development of various mutant yeast collections and automated screening techniques allows for fast, high-throughput analysis of large numbers of compounds with high target pathway resolution. Typically, such screens analyze the chemical sensitivity of arrayed libraries of yeast mutants, in which each mutant has an altered dosage of a specific gene [49]. Gene dosage may range from no expression (the yeast deletion collection [7,8]) or very low gene expression (by decreasing mRNA stability due to deletion of the 3' UTR, also termed DaMP, for decreased abundance by mRNA perturbation [56]), to a mild reduction in gene expression levels (haploid insufficiency libraries, in which one of the two copies of a gene in a diploid strain has been deleted [57]), or very high gene expression (gene overexpression libraries [58]).

Chemical profiling can be performed in several ways. Yeast collections can be spotted on plates harboring the drug of interest, and numerous drugs have been profiled using this method. For instance, pathways that mediate resistance to the DNA methylating agent MMS have been mapped this way [59]. An alternative screening method relies on competitive growth assays of pooled yeast cultures (each pooled culture consists of a mix of known mutants). Several yeast libraries, including the deletion collection, have been barcoded such that each mutation harbors a unique sequence tag, which can be identified by hybridization methods or high-throughput sequencing. By growing pooled cultures of yeast mutants in the presence of the drug of interest, one can identify mutations that confer a relative growth advantage or disadvantage [15,57]. This method is sensitive and quantitative; for example, it revealed additional genes required for resistance to MMS that were not identified in the previously mentioned high-throughput screen for MMS-sensitive mutants [60]. The great advantage of analyzing pooled cultures of barcoded mutants is that large numbers of drugs can be screened simultaneously at various concentrations and in different combinations [61]. 

Several bioinformatics tools have been developed to facilitate the identification of the mode of action of a given bioactive compound. A major tool in determining a drug’s mechanism of action is clustering analysis. First, the entire set of haploid yeast deletion mutants is treated with a compound of interest and the response of all deletion mutants is documented (usually growth rates are monitored). Then, all mutants that display similar behavior in response to the compound are assigned into clusters, thereby generating a specific chemical-genetic profile; this profile is predictive of the drug’s mechanism of action. The search for a molecular target can be further narrowed by comparing the chemical-genetic profile of the drug to genetic interaction profiles of loss-of-function mutants. The reasoning behind this is that a loss-of-function mutation in a gene encoding the target of an inhibitory compound models the primary effect of the compound , and therefore the genetic interaction profile for the target gene resembles the chemical-genetic interaction profile of its inhibitory compound [62]. Thus, one can infer the molecular target of a drug by matching the drug’s chemical-genetic profile to the genetic interaction profile of loss-of function mutants [63]. This method is particularly powerful in budding yeast, because the genetic interaction profiles of the vast majority of yeast genes are known [20].

However, these high-throughput drug profiling screens also show that drugs often display a much more complex behavior in a living cell than expected from *in vitro* determination of a drug’s biochemical activity [64]. One complication of these studies is that genes involved in cellular detoxification and general stress response are commonly identified (multidrug resistance genes), adding undesirable noise to the dataset that makes determination of the drug’s mode of action less straightforward. Filtering these genes before clustering analysis may help remove this noise [62]. Furthermore, some drugs still have an effect on cellular fitness even when the drug target is no longer expressed [65], although determining these off-target effects can give important information with potential clinical relevance [66]. Finally, a recent, very large scale chemical-genomics screen revealed that nearly all yeast genes are important for cell fitness, even though these functions are not easily detectable in standard laboratory settings [61]. This study also revealed a large set of genes involved in multidrug resistance, such as genes encoding the transcription factor *PDR1*, which is involved in transcription of multidrug resistance genes such as the pleiotropic drug pump *PDR5*. Other multidrug resistance genes were involved in regulation of membrane lipid composition and vesicular transport of proteins. 

Due to these issues, not all high-throughput screens in haploid deletion collections may reveal the mechanism of action of a drug. However, these studies still provide insight in cellular strategies against chemical stress [67], and integration of data from several high-throughput studies has revealed novel levels of complexity. For instance, recent studies have obtained large collections of chemical-genetic drug profiles, and integration of these data with information from other high-throughput analyses, such as kinase-substrate networks, protein-protein complexes, and structure and occupancy of promoters, revealed several underlying buffering mechanisms that promote cell survival during chemical stress [68,69]. For further reading on the development and applications of chemical-genomic profiling in budding yeast, the reader is referred to several recent reviews [64,70,71,72,73].

## 6. Conclusions and Future Directions

From the initial isolation of pure budding yeast strains from wort in the late 1800s to modern high-throughput automated genetic screens, yeast genetics has provided insight in numerous biological processes, including regulation of the cell cycle, autophagy, cell polarity, and repair of DNA damage. Recent developments in the field of chemical-genetics have provided tools to further dissect the genetic network of the cell, and yeast will be the first eukaryotic organism in which all genetic pathways have been mapped. Furthermore, studies in budding yeast will have clinical relevance, because chemical-genetic screens and chemical-genomic profiling are becoming increasingly important tools for drug discovery.

By far most of the genetic interaction studies have been performed under optimal growth conditions. However, under conditions of cell stress, the cell may rely on very different genetic pathways to promote cell viability, and it may rewire its biological networks to deal with the altered conditions. For example, *HSP90*, which encodes the major molecular chaperone in yeast, genetically interacts with very sets of genes under normal growth conditions than when cells are challenged with environmental stress [74]. This is also observed in cancer cells, which rewire their signaling networks in response to extracellular signals, such as cytokines and anti-cancer drugs, with important implications for cancer treatment schemes [75,76,77]. Understanding how cells dynamically rewire their networks during environmental challenges will be a major subject of future studies.
